# Supplementary figures and images for: Evaluation of the Diagnostic Performance of Two Automated SARS-CoV-2 Neutralization Immunoassays following Two Doses of mRNA, Adenoviral Vector, and Inactivated Whole-Virus Vaccinations in COVID-19 Naïve Subjects
Source: Microorganisms. 2023 Apr 30;11(5):1187. doi: 10.3390/microorganisms11051187 (PMC10220828; doi:10.3390/microorganisms11051187)

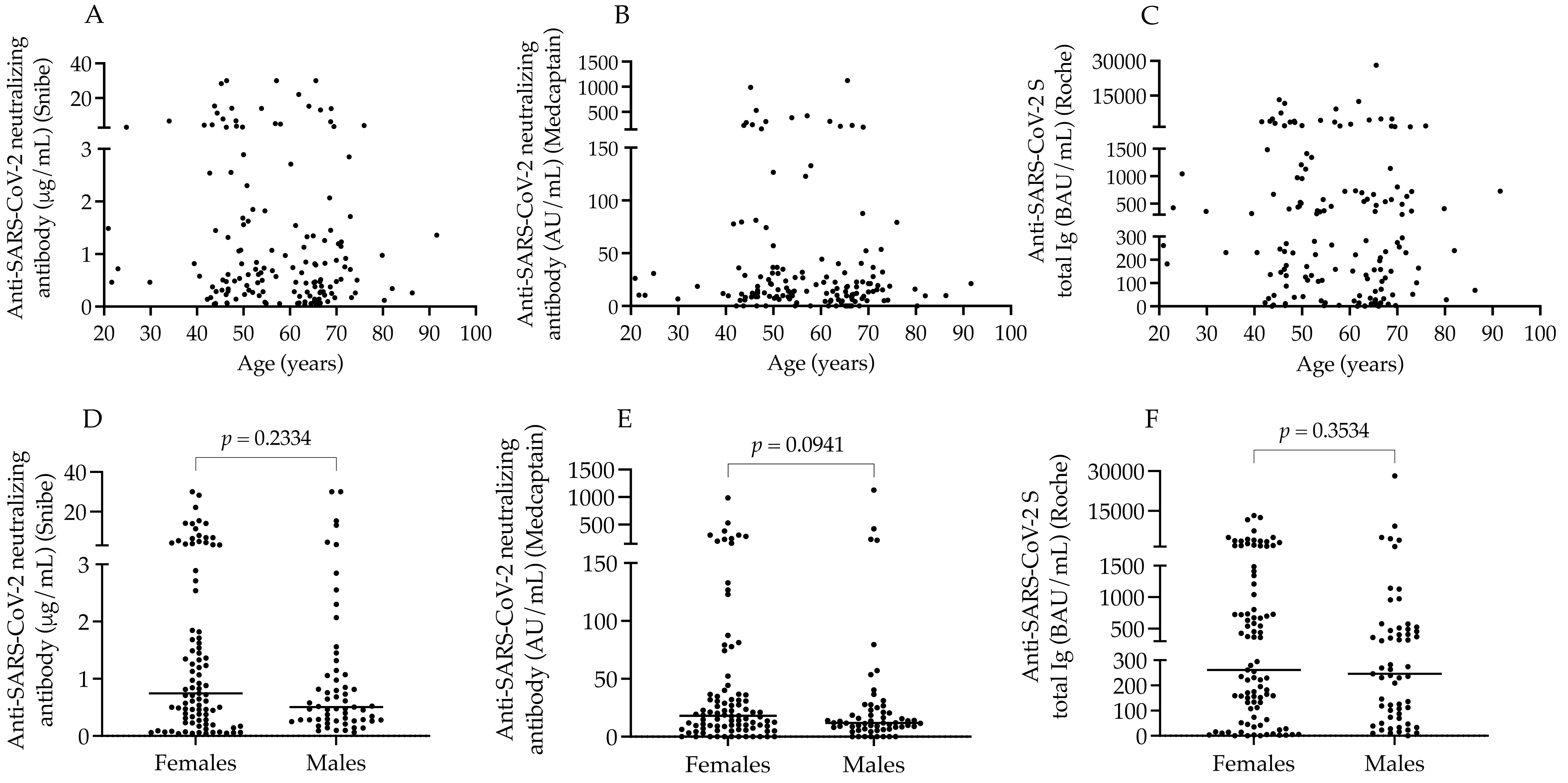

Supplement: Supplementary file 1 [file microorganisms-11-01187-s001.zip › microorganisms-2299330-supplementary.tif]
